# Autoimmune/Inflammatory Syndrome Induced by Hyaluronic Acid and Hyaluronidase

**DOI:** 10.7759/cureus.111216

**Published:** 2026-06-20

**Authors:** Olga Vera-Lastra, Abihai Lucas-Hernandez, Elsa Acosta-Jiménez, Ana L Peralta-Amaro, Gabriela Medina, Maria del Pilar Cruz-Dominguez, Yehuda Shoenfeld

**Affiliations:** 1 Department of Internal Medicine, Hospital de Especialidades Dr. Antonio Fraga Mouret, Instituto Mexicano del Seguro Social, Mexico City, MEX; 2 Department of Rheumatology, Hospital General de Teziutlán, Servicios de Salud del Instituto Mexicano del Seguro Social para el Bienestar (IMSS-Bienestar), Teziutlán, MEX; 3 Department of Pathology, Hospital de Especialidades Dr. Antonio Fraga Mouret, Instituto Mexicano del Seguro Social, Mexico City, MEX; 4 Department of Postgraduate Education, Universidad Nacional Autónoma de México, Mexico City, MEX; 5 Translational Research Unit, Hospital de Especialidades Dr. Antonio Fraga Mouret, Centro Médico Nacional La Raza, Mexico City, MEX; 6 Direction of Research and Education, Hospital de Especialidades Dr. Antonio Fraga Mouret, Centro Médico Nacional La Raza, Mexico City, MEX; 7 Department of Internal Medicine, Zabludowicz Center for Autoimmune Diseases, Sheba Medical Center, Ramat Gan, ISR; 8 Faculty of Medicine, Reichman University, Herzliya, ISR

**Keywords:** autoimmune/inflammatory syndrome induced by adjuvants (asia), cutis laxa, delayed hypersensitivity reaction, hyaluronic acid, hyaluronidase

## Abstract

Skin aging is a biological and chronological process characterized by progressive alterations in the extracellular matrix, including reduced hyaluronic acid (HA) content. HA dermal fillers are widely used for cosmetic purposes and are generally considered safe; however, immune-mediated complications, including autoimmune/inflammatory syndrome induced by adjuvants (ASIA), have been increasingly reported. We report the case of a 35-year-old White woman who underwent cosmetic lip augmentation with HA and subsequently developed multisystem manifestations consistent with ASIA. Due to persistent local and systemic symptoms, hyaluronidase was administered; however, her condition deteriorated, with progression to systemic involvement. Clinical features included sicca symptoms, Raynaud’s phenomenon, and cognitive impairment with reduced concentration and memory, severe fatigue, and progressive cutaneous changes characterized by skin laxity and hyperextensibility, compatible with acquired cutis laxa. Despite treatment with high-dose corticosteroids, methotrexate, analgesics, and antidepressants, no significant clinical improvement was observed. This case highlights the potential for severe immune-mediated and connective tissue complications following exposure to HA and hyaluronidase. Awareness of ASIA and its atypical manifestations, including acquired cutis laxa, is essential for early recognition and appropriate management of patients undergoing cosmetic procedures.

## Introduction

Skin aging is a complex biological process influenced by genetic predisposition, environmental factors, and lifestyle. These processes are associated with a progressive reduction in the synthesis and structural integrity of collagen, elastin, and hyaluronic acid (HA), which are essential for maintaining skin firmness, elasticity, and hydration. Consequently, the pharmaceutical and cosmetic industries have developed a wide range of anti-aging interventions. HA is a naturally occurring glycosaminoglycan and a key component of the extracellular matrix. Owing to its minimal immunogenicity, high biocompatibility, and excellent hydrophilic properties, HA is among the most widely used substances for aesthetic dermal fillers [1]. Although HA dermal fillers are generally considered safe, the incidence of severe and undesirable complications has increased with the growing number of procedures performed worldwide. In 2019 alone, more than five million HA filler procedures were reported by the American Society of Plastic Surgeons and the American Society for Dermatologic Surgery [2]. Adverse events related to HA fillers are commonly classified as early (occurring within days to months) or late (occurring months to years after injection). Early complications include bruising, edema, hematoma, hypersensitivity reactions, asymmetry, infections, and vascular compromise, which may lead to severe outcomes such as vision loss. Late complications include foreign body granuloma formation, delayed hypersensitivity reactions, Tyndall effect, chronic infections, and other immune-mediated phenomena [3].

Hyaluronidase, an enzyme that degrades HA into monosaccharides, is widely used as an antidote for complications associated with HA fillers. Although highly effective, hyaluronidase is not without risks. Allergic and inflammatory reactions, including urticaria, angioedema, and anaphylaxis, have been reported, particularly in individuals previously sensitized by insect stings or prior exposures [4]. Although HA is generally considered biocompatible, increasing evidence suggests that, under certain conditions, it may act as an immune adjuvant and contribute to the development of immune-mediated manifestations in susceptible individuals [5,6]. Autoimmune/inflammatory syndrome induced by adjuvants (ASIA) is diagnosed based on a combination of clinical and exposure-related criteria, including prior exposure to a potential adjuvant, the development of characteristic systemic manifestations, and compatible histopathological findings. According to the updated classification, the diagnosis is supported when either two major criteria or one major and two minor criteria are fulfilled [7]. Nevertheless, the diagnosis of ASIA remains challenging because many of its clinical manifestations are nonspecific and may overlap with those of autoimmune, inflammatory, neurological, and functional disorders.

Acquired cutis laxa is a rare connective tissue disorder characterized by progressive loss of skin elasticity due to alterations of elastic fibers and extracellular matrix remodeling. Although it has been associated with autoimmune and inflammatory conditions, its occurrence in the context of ASIA related to HA exposure has rarely been reported [8].

Given the expanding use of HA fillers worldwide, recognition of potential immune-mediated systemic complications is increasingly relevant for rheumatologists and immunologists. The aim of this report is to describe a case of ASIA temporally associated with exposure to HA and subsequent administration of hyaluronidase, accompanied by multisystem manifestations and clinical and histopathological findings compatible with acquired cutis laxa.

## Case presentation

This is a 35-year-old woman with no known family history of autoimmune diseases, connective tissue disorders, or cutis laxa. Her past medical history was notable for allergic rhinitis, with documented allergies to pollen, dust, cat hair, and peanuts. She had no prior history of food intolerance. Following exposure to HA, she developed new-onset intolerance to dairy and gluten.

In June 2024, she underwent cosmetic lip augmentation with a single injection of HA filler (Stylage® L). Notably, she had previously received a lip filler procedure with Stylage® M approximately two years earlier without adverse reactions. Two days after the procedure, she developed progressive neurological and mucocutaneous symptoms, including numbness and tingling of the face, gums, and scalp, insomnia, anxiety, impaired concentration and memory, visual disturbances, painful lip nodules, oral blisters, and erythematous patches on the tongue. Because the symptoms persisted and worsened, hyaluronidase (Hyalase®) was administered on four occasions in July 2024. Detailed information regarding the dose and dilution was not available because the procedure was performed at an outside cosmetic clinic. Immediately after the second administration, the patient experienced acute facial paresthesias, dyspnea, and palpitations. Subsequently, her clinical condition worsened, with the development of sleep disturbances, gingival nodules, persistent headache, alopecia involving the scalp and eyelashes, and urinary symptoms. Over the following months, she reported recurrent episodes of severe fatigue associated with multisystem involvement, including neurological, cutaneous, and musculoskeletal manifestations. Additional symptoms included xerophthalmia, xerostomia, Raynaud’s phenomenon, neuropathic pain, cognitive dysfunction, and progressive skin changes characterized by increased skin laxity and loss of subcutaneous tissue, particularly around the joints (Figure 1). Musculoskeletal manifestations included pain in large joints and diffuse myalgia, predominantly affecting the cervical and dorsal regions, without overt inflammatory features.

**Figure 1:**
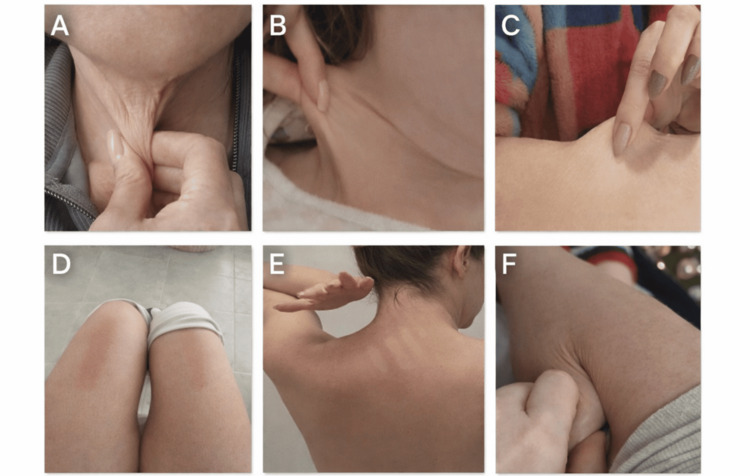
Cutaneous manifestations following hyaluronidase exposure Representative clinical photographs illustrating cutaneous changes observed after hyaluronidase administration. (A-C) Marked skin laxity and hyperextensibility involving the cervical region and trunk, demonstrated by increased distensibility and reduced elastic recoil upon manual traction. (D) Linear erythematous lesions on the thighs. (E) Linear erythematous lesions on the upper back. (F) Pronounced skin hyperlaxity of the lower extremity, with apparent loss of subcutaneous tissue support.

On physical examination, vital signs were within normal limits. The patient was alert and oriented but appeared anxious. Conjunctival hyperemia and signs of oral dryness were noted. Cardiopulmonary examination was unremarkable. Several large and small joints were tender to palpation; however, no joint swelling, erythema, increased local temperature, or other clinical signs of synovitis were observed. Marked skin hyperlaxity was observed, predominantly in the neck and extremities, with increased extensibility, reduced elastic recoil, and apparent loss of subcutaneous tissue, findings raising suspicion for acquired cutis laxa (Figure 1). Peripheral arterial pulses were preserved.

Extensive laboratory evaluation, including a complete blood count, serum chemistry, electrolytes, hepatic and renal function tests, lipid profile, thyroid function tests, antinuclear antibodies, anti-double-stranded DNA, anti-Ro, anti-La, and anti-Sm antibodies, rheumatoid factor, anti-citrullinated peptide antibodies, C-reactive protein, complement C3 and C4 levels, and serum protein electrophoresis, revealed no clinically significant abnormalities. Extensive autoimmune evaluation did not support the diagnosis of a defined systemic autoimmune rheumatic disease, including systemic lupus erythematosus (SLE), Sjögren’s disease (SD), rheumatoid arthritis (RA), or other connective tissue disorders. Brain magnetic resonance imaging (MRI) revealed three small hyperintense foci on T2-fluid-attenuated inversion recovery (FLAIR) sequences within the right frontal subcortical white matter, measuring up to 4 mm in diameter. These findings were considered nonspecific based on their appearance and location (Figure 2).

**Figure 2:**
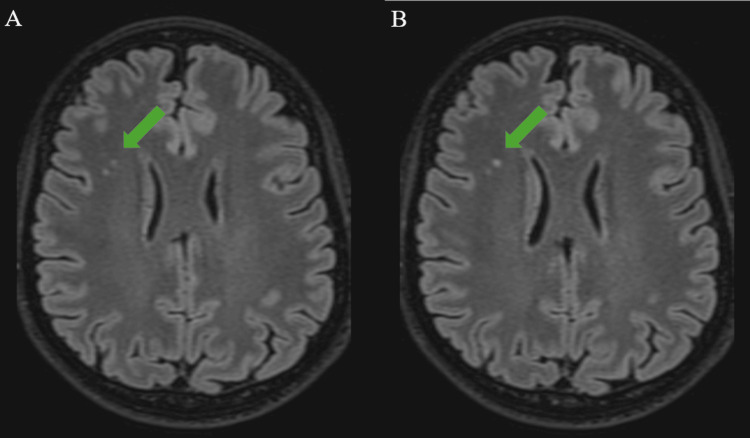
Brain magnetic resonance imaging findings (A, B) Axial T2-fluid-attenuated inversion recovery (FLAIR) brain magnetic resonance imaging demonstrating small hyperintense foci within the right frontal subcortical white matter (largest measuring approximately 4 mm) (arrows). These lesions were nonspecific in appearance and location, and their clinical significance remains uncertain.

Skin biopsy demonstrated preserved epidermal architecture and dermal laxity with mild perivascular lymphocytic inflammatory infiltrates, focal areas of dense collagen deposition, and marked separation and fragmentation of elastic fibers demonstrated on Verhoeff elastic stain, findings consistent with acquired cutis laxa (Figure 3).

**Figure 3:**
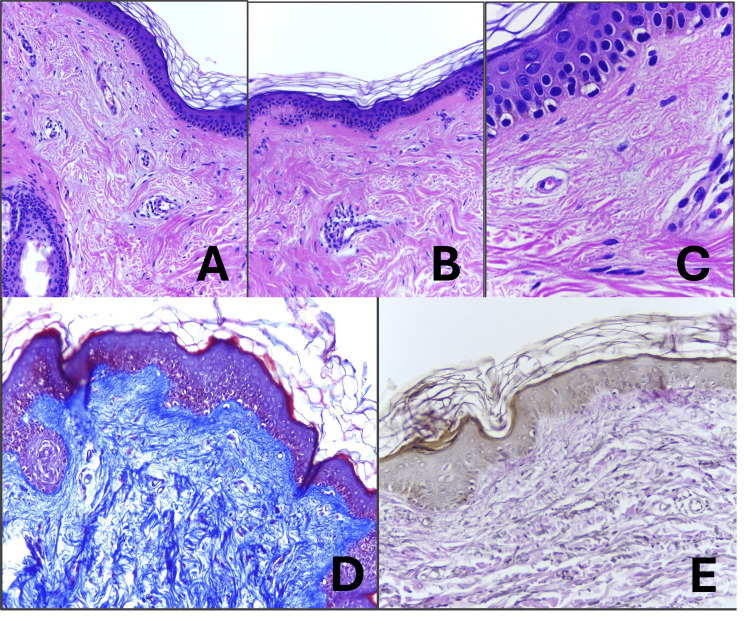
Histopathological findings compatible with acquired cutis laxa (A-B) Low-power panoramic view of the skin showing an unremarkable epidermis. The dermis demonstrates a lax appearance with a mild perivascular lymphocytic infiltrate and focal areas of denser collagen deposition, which may mimic hypertrophic scar formation. (C) Higher magnification view of the areas with dermal laxity. (D) Masson’s trichrome stain highlighting increased separation of elastic fibers in the central area. (E) Verhoeff elastic stain demonstrating generalized fragmentation and disruption of elastic fibers, findings compatible with acquired cutis laxa.

Given the temporal relationship between HA exposure and subsequent development of multisystem manifestations, the patient met three major and one minor criteria for ASIA according to the updated classification, supporting the diagnosis (Table 1).

**Table 1:** Clinical findings supporting the diagnosis of ASIA Adapted from Cohen Tervaert et al. [7]. Based on these criteria, the patient fulfilled three major criteria and one minor criterion, supporting the diagnosis of ASIA. ASIA: autoimmune/inflammatory syndrome induced by adjuvants

ASIA criterion	Findings in this patient
Major criterion: exposure to an external stimulus (adjuvant) prior to clinical manifestations	Cosmetic lip augmentation with hyaluronic acid (Stylage® L) followed by four administrations of hyaluronidase
Major criterion: appearance of typical clinical manifestations	Chronic fatigue, sleep disturbances, myalgia, arthralgia, cognitive impairment, sicca symptoms, and neurological manifestations
Major criterion: typical biopsy of involved organs	Skin biopsy showing mild perivascular lymphocytic inflammatory infiltrates. In addition, dermal laxity and elastic fiber fragmentation, as demonstrated by Verhoeff stain, were observed, supporting a concurrent diagnosis of acquired cutis laxa
Minor criterion: other clinical manifestations	Raynaud’s phenomenon, alopecia, mucocutaneous manifestations, neuropathic symptoms, and urinary symptoms

Initial symptomatic treatment included pregabalin, analgesics, anti-inflammatory drugs, antihistamines, low-dose naltrexone (1 mg in the morning and 0.75 mg in the evening), vitamin D3, and omega-3 fatty acids. Because symptoms persisted, she subsequently received prednisone (initially 30 mg/day, gradually tapered to 5 mg/day, which she continues at present), intravenous methylprednisolone pulses (1 g daily for three consecutive days), hydroxychloroquine for approximately eight months, and methotrexate, initially at 15 mg weekly and currently maintained at 10 mg weekly. Despite these interventions, the patient has continued to experience persistent fatigue, cognitive symptoms, sicca manifestations, musculoskeletal pain, and progressive cutaneous changes, without significant clinical improvement during follow-up. The overall clinical course is summarized in a timeline (Figure 4).

**Figure 4:**
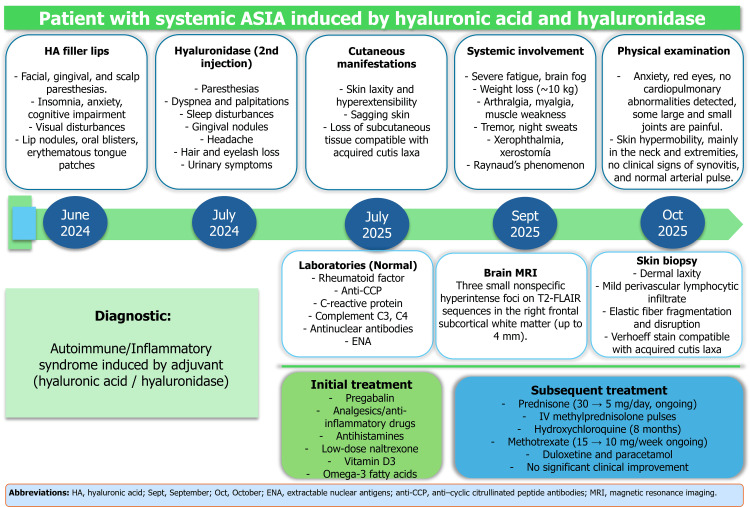
Timeline of clinical course in a patient with systemic autoimmune/inflammatory syndrome induced by adjuvants (ASIA) induced by hyaluronic acid and hyaluronidase The timeline summarizes symptom progression, diagnostic findings, and therapeutic interventions from initial exposure through follow-up.

## Discussion

This case illustrates a complex clinical presentation temporally associated with exposure to HA and subsequent administration of hyaluronidase. The patient developed multisystem manifestations fulfilling the diagnostic criteria for ASIA and progressive cutaneous changes that were clinically and histopathologically compatible with acquired cutis laxa. ASIA is defined by immune-mediated manifestations following exposure to an external stimulus with adjuvant properties, including vaccines, silicone, mineral oil, methyl methacrylate, and, more recently, HA. The diagnostic criteria, first proposed by Shoenfeld in 2011, emphasize a temporal relationship between exposure and symptom onset, the development of typical clinical manifestations, and the exclusion of alternative diagnoses [6]. In this case, the temporal association between HA injection and the onset of neurological, neuropsychiatric, mucocutaneous, and musculoskeletal symptoms supports the diagnosis; however, a definitive causal relationship cannot be established based on a single case report.

Alternative explanations for the patient’s multisystem manifestations were carefully considered. Extensive laboratory and autoimmune evaluation did not support the diagnosis of a defined systemic autoimmune rheumatic disease, including SLE, SD, RA, or other connective tissue disorders. Likewise, no alternative neurological, inflammatory, or metabolic condition was identified to account for the combination of neurological, mucocutaneous, musculoskeletal, and constitutional symptoms. Although some manifestations, such as fatigue, cognitive complaints, and sicca symptoms, are nonspecific and may occur in a variety of clinical settings, the temporal relationship between exposure to HA, subsequent hyaluronidase administration, and the progressive development of multisystem manifestations supports the consideration of an immune-mediated process. Nevertheless, a definitive causal relationship cannot be established based on a single case report.

HA is generally regarded as biologically inert and minimally immunogenic; however, growing evidence suggests that, under certain conditions, it may act as an immune adjuvant. Experimental and clinical data indicate that HA fragments can interact with pattern recognition receptors, including Toll-like receptors, thereby activating innate immune pathways and releasing proinflammatory cytokines, ultimately leading to sustained immune stimulation [6,9]. These mechanisms may explain the broad systemic manifestations observed in susceptible individuals.

In this case, symptom exacerbation following hyaluronidase administration is a significant clinical observation. Although widely used as an antidote for HA filler-related complications, hyaluronidase is not devoid of immunologic risk. Immediate hypersensitivity reactions and delayed inflammatory responses, including urticaria, angioedema, and, rarely, anaphylaxis, have been reported [4,5]. Prior sensitization from insect stings or repeated exposures has been identified as a potential risk factor for allergic reactions to hyaluronidase [4,9-11]. In this patient, the acute onset of neurological and respiratory symptoms immediately after the second dose suggests an immune-mediated reaction that may have amplified the underlying inflammatory process.

A particularly striking feature of this case is the development of cutaneous manifestations compatible with acquired cutis laxa. Acquired cutis laxa is a rare connective tissue disorder characterized by progressive loss of skin elasticity due to damage to elastic fibers and remodeling of the extracellular matrix, often associated with inflammatory, autoimmune, infectious, or drug-induced mechanisms. In this patient, the combination of progressive skin laxity, loss of subcutaneous tissue, and histopathological findings supports this diagnosis. Alternative diagnoses were considered. Hereditary forms of cutis laxa were considered unlikely because of the absence of a personal or family history of connective tissue disease and the adult onset of symptoms. Ehlers-Danlos syndrome was also considered less likely given the lack of a previous history of skin hyperextensibility or other characteristic connective tissue manifestations. Similarly, anetoderma, mid-dermal elastolysis, pseudoxanthoma elasticum, and corticosteroid-related atrophy were considered less compatible with the diffuse clinical presentation and the histopathological findings, particularly the fragmentation and disruption of elastic fibers demonstrated on Verhoeff stain. Although acquired cutis laxa has been described in association with autoimmune diseases and inflammatory triggers, its occurrence following hyaluronidase exposure remains exceedingly rare and underscores the novelty of this case [10,11]. Neurological involvement, including cognitive impairment, has been increasingly reported in ASIA-related conditions, supporting central nervous system involvement in this entity [5,6].

## Conclusions

To our knowledge, this appears to be the first reported case of multisystem manifestations fulfilling diagnostic criteria for ASIA temporally associated with exposure to both HA and hyaluronidase, accompanied by clinical and histopathological findings compatible with acquired cutis laxa. This case highlights the importance of considering possible immune-mediated reactions in patients presenting with unexplained multisystem symptoms following cosmetic procedures involving HA and subsequent hyaluronidase administration. Although causality cannot be established from a single case report, clinicians should be aware of this potential association and consider appropriate clinical evaluation and follow-up. Early recognition may help avoid repeated exposure to potential triggers and facilitate multidisciplinary management.
